# Realism and the point at infinity: The end of the line?

**DOI:** 10.1007/s11229-023-04228-w

**Published:** 2023-08-29

**Authors:** Oisín Parkinson-Coombs, Rafael Núñez

**Affiliations:** Cognitive Science, UCSD, Gilman Drive, La Jolla, CA 92092 USA

**Keywords:** Projective geometry, Cognitive linguistics, Realism, Mathematical progress, Infinity, History

## Abstract

Philosophers of mathematics often rely on the historical progress of mathematics in support of mathematical realism. These histories typically build on formal semantic tools to evaluate the changes in mathematics, and on these bases present later mathematical concepts as refined versions of earlier concepts which are taken to be vague. Claiming that this view does not apply to mathematical concepts in general, we present a case-study concerning projective geometry, for which we apply the tools of cognitive linguistics to analyse the developmental trajectory of the domain. On the basis of this analysis, we argue for the existence of two conceptually incompatible inferential structures, occurring at distinct moments in history, both of which yield the same projective geometric theorems; the first invoked by the French mathematicians Girard Desargues (1591–1661) and Jean-Victor Poncelet (1788–1867), and the second characterising a specific modern mode. We demonstrate that neither of these inferential structures can be considered as a refinement of the other. This case of conceptual development presents an issue to the standard account of progress and its bearing on mathematical realism. Our analysis suggests that the features that distinguish the underlying conceptually incompatible inferential structures are invisible to the standard application of the tools of formal semantics. Thus this case-study stands as an example of the manner and necessity of linguistics—specifically cognitive linguistics—to inform the philosophy of mathematics.

## Introduction

### Realism and progress

In an address delivered at a public meeting of the Royal Academy of Sciences of Gottingen in 1895 Felix Klein stated that

[arithmetised analysis] leads us back to what is more nearly the geometry of the ancients, and in the light of our modern ideas we learn to understand precisely the *true nature* of [the geometry of the ancients]’ (Klein, 1895, p. 244, emphasis added).

Similarly, mathematician and educator Hans Freudenthal opens his essay on algebra and its history stating

‘whoever starts reading Greek mathematics is struck by large parts that are *overtly algebraic* as well as other parts where *algebra seems to hide* under a geometrical cover’ (Freudenthal, 1977, p. 189, emphasis added).

These historiographies of mathematics from eminent mathematicians implicitly articulate a very specific account of mathematics and its progress. In order to have a *true nature*, or to *hide*, a concept must ostensibly exist independently of the mathematics of that time; the concept’s *hidden* features are what made their mathematics work. This specific account, although perhaps grounded in these mathematicians’ situated introspections, is not unique to them. Nicholson, detailing the history of the quotient group, describes Galois’ ‘implicit use of the concept’ (Nicholson, 1993, p. 70) and writes

‘with the benefit of hindsight we can see that the *concept of quotient group was present* on many occasions before an explicit definition was given’ (Nicholson, 1993 , p. 70, emphasis added).

These statements reflect a pre-theoretic mathematical realism. This entails that mathematics, and its development, is uniquely determined by the features of these as yet unexplicated concepts. This realist belief requires substantiation.

Some philosophers of mathematics have addressed the historical development of mathematical knowledge and its relation to mathematical realism (Hafner & Mancosu, 2008; Kitcher, 1988; Liston, 2000; Manders, 1989). These authors use formal semantics, with differing degrees of nuance and historicism, to evaluate the changes that occur throughout the development of mathematical concepts. For instance, Michael Liston explores the development of summable infinite series, claiming it to be a ‘mathematical development that exemplifies progress’ (Liston, 2000, p. 257), and its relation to Hilary Putnam’s *no-miracles argument*^1^ (Putnam, 1975). Liston focuses on the change from

- Euler—relying on the principle that ‘the “sum” of an infinite series is the finite, algebraic expression that generates the series’ (Liston, 2000, p. 258),
- to Cauchy—introducing a strict convergence constraint to ground the summable series and legitimate their arithmetic operations
- to Fröbenius, Holder, and others—attempting to construct a foundation for summable series that would explain the consistency in ascribing sums to some divergent series as well as convergent series.

This case-study is immediately concerned with the changes in the summability conditions of the infinite series and, the attempts of mathematicians to justify and explain their successes and limitations: the different ways of fixing the meaning of the sums, and how these legitimate their use. These features of this case-study encourage a reliance on ‘truth-reversals’—e.g. the alternating summability evaluations in different eras—and ‘hidden tensions’—e.g. Bernoulli’s concern that Euler’s sums ‘might not be unique, since the series’ generating function might not be unique’ (Liston, 2000, p. 261)—within a concept and its application as a means to analyse the development of the concept.

These technical tools are especially useful in analysing mathematical developments that are driven by endogenous issues and their solutions, issues generated by contradictions or confusions within the mathematical concept, its inferential structure,^2^ and the results of its application. Liston argues that, with respect to the eventual formal definitions of summability, ‘though these conditions received explicit articulation only from the 1880s on, it is clear that they *lay in the background* of most work on infinite series’ (Liston, 2000, p. 260, emphasis added), articulating and substantiating a position similar to those historical positions above. Similarly Kitcher, in a brief case-study concerning Viete and Lagrange’s systematization by conceptualisation of the solutions to cubics states that ‘new language enables us to perceive *the common thread* which runs through our old problem solutions, thereby increasing our insight into *why those solutions worked*’ (Kitcher, 1984, p. 221, emphasis added). On these accounts, and through these analytic tools, conceptual change is claimed to be continuous—which is an important requirement for the *no-miracles argument* to hold. The truth-conditional changes, which one might initially view as evidence of discontinuities in the concept, are argued to arise from tensions within the original concept, which are evaluated using similar tools: the concept itself was initially vague, and mathematicians have refined our understanding of this fixed concept by exploring its ‘hidden tensions’.

Our point here is not that these case studies are ahistorical, or use inadequate tools. Nor are we attempting to claim that realism—which we take to be the general commitment that ‘(1) the sentences of that theory or discourse are true or false; and (2) that what makes them true or false is something external—that is to say, it is not (in general) our sense data, actual or potential, or the structure of our minds, or our language, etc.’ (Putnam, 1975, p. 70)—is necessarily incorrect. Rather we aim to highlight the features of these case-studies that make them amenable to a formal semantic analysis, and thus to stand as a point of contrast to the features of the case-study that we later develop here. Further, we highlight the import of these case-studies with respect to mathematical realism, by exemplifying the means by which cognitive linguistic tools can be meaningfully brought to bear on philosophical debates: namely, by introducing diversity of data and paradigms of mathematical progress, against which the strength of the arguments for realism (e.g. the *no-miracles argument*) can be judged.

### Central argument and article structure

In this article, we introduce a case-study of the development of projective geometry to demonstrate that not all cases of mathematical progress can be adequately explained by a focus on ‘truth reversals’ or ‘hidden tensions’. Using the tools of cognitive semantics and linguistics, broadly understood, we analyse the development of this domain from its inception in the 17th century up to its modern characterisation. On the basis of this case-study we identify two distinct inferential modes. These inferential modes are theoretical descriptions of the manner in which inference is performed, and accepted, by mathematicians. In this case-study, these distinct inferential modes yield conceptually incompatible inferential structures for projective geometry, however the theorems of these incompatible inferential structures are the same.

The first such mode we call the **local** ad hoc mode which characterises the pre-modern approach as well as some modern mathematical practice. This mode exhibits context-dependent constructions of the point at infinity—which we will later analyse using frame semantics—which are not substitutable for one another in inference. The second mode we term the **global unitary** mode, typifying modern mathematics. This mode consists of a monolithic inferential structure in which any construction of the point at infinity must be substitutable for any other such construction, regardless of the manner in which the representation is constructed. The differences between these two modes of inference will be described and made specific using the tools of cognitive linguistics. Further, we will use a central cognitive conflict of projective geometry (often mentioned in textbooks) as a probe to highlight the incompatibility of the inferential structures developed in these distinct modes of inference, where by conceptual incompatibility we mean that some of the main concepts of one inferential structure cannot be expressed in the terms of the other.

We argue that the changes from the pre-modern to the modern inferential structure are not the results of ‘hidden tensions’ or endogenous issues, but that these changes are imposed due to a shift in metamathematical views developed in different domains of mathematics, notably

- a shift in the sanctioned mode of inference from the local to the global,
- and a requirement for the projective geometry to preserve and extend some features of formalised Euclidean geometry.

These changes cannot be evaluated by ‘truth reversals’ and formal descriptions of the two inferential structures, because the projective geometric truth statements are the same in both. The earlier and later stages of development present two alternative foundations for projective geometry, and these alternative foundations provide conceptually incompatible inferential structures, neither can be simply accepted as an imprecise version of the other. Thus, while mathematical realist claims concerning ‘common threads’, ‘lying in the background’ etc. may be appropriate for certain paradigms of mathematical development, the results of detailed cognitive linguistic analysis of this case-study raise the prospect that not all mathematical developments can be described in such a fashion. As such, this paper stands as an example of how linguistics, specifically cognitive linguistics, can inform the philosophy of mathematics by extending the domain of relevant data and revealing ostensibly equivalent mathematical systems but with conceptually incompatible inferential structures.

With respect to conceptual continuity, and its relation to mathematical progress and realism, we must first mention two theoretical choices upon which the arguments may turn:

- the level at which continuity is adjudicated: one may be concerned with the continuity of problems, or the continuity of solutions as the relevant locus for realist claims
- which features one accepts as mathematical or metamathematical: whether a change during development in the problem or solution arises from a feature judged mathematical or metamathematical might alter the judgement of (dis)continuity.

In this paper we address neither of these questions, as they are beyond the scope. We accept standards of proof (or sanctioned modes of inference) as metamathematical, in keeping with Kitcher (1984), and we show that these metamathematical constraints alter the problem-space and the corresponding solutions. These choices and perspectives may rely on philosophical commitments that we cannot resolve or investigate here. With respect to these philosophical positions we offer our case-study, and our analysis of it, as an example of cognitive linguistic methods, the data and the results that they can bring to bear on history and philosophy of mathematics.

We first introduce some tools of cognitive linguistics that we will rely on in our case-study analysis in Sect. 2. We will then introduce projective geometry and its central cognitive conflict in Sect. 3. We analyse mathematical reference texts, mathematical textbooks, and the seminal texts of the field to understand the inferential systems evidenced in these texts. On the basis of this analysis we describe the two conceptually incompatible inferential structures mentioned above, and how these could bear on mathematical development in Sect. 4. Finally, we relate this case-study and its findings to the philosophies of mathematics to argue for the need to expand our analytic tools beyond formal semantics and to include cognitive linguistics in Sect. 5.

## Cognitive semantic preliminaries

### Cognitive semantic methods

Cognitive semantics, is a sub-field of cognitive linguistics, an area of linguistics that emerged in the 1980s, primarily with the work of linguists such as Charles Fillmore (1982), Ron Langacker (1987), Len Talmy (1988, 1996), George Lakoff (1982, 1987), Gilles Fauconnier (1985, 2018), among others. Focusing on the study of the ordinary functions of language and performing detailed and careful analyses of actual everyday linguistic expressions, scholars in cognitive linguistics have argued that language emerges from general cognitive mechanisms, rather than from an autonomous domain-specific language faculty. A central idea is that by studying speakers’ construals as primitives—the actual use and sense-making that is brought forth via linguistic expressions (whether they are grammatical or not; literal or metaphorical, etc.)—it is possible to investigate deep aspects of people’s conceptualisations and cognition, which themselves may be pre- or non-linguistic. This approach differs in fundamental ways from that of formal semantics which typically takes as primitives elements such as: truth conditions, a priori defined conditions of satisfaction; the syntactic rules in sentences that combine the alleged meanings of subjects with the alleged meanings of predicates; and even takes as starting point the very formal language needed for the treatment of meaning with its discrete symbols and syntactic rules for compositionality (e.g., $(\forall x)(\exists y) \phi (x, y)$ to formally express *‘Everything bears the relation* $\phi$ *to at least one thing’*). One prolific area of research in cognitive semantics is that of abstraction and imagination, such as those that via conceptual metaphor allow, for instance, the study of spatial construals of time as manifested in the English expressions *‘She left her past behind’* and *‘The week ahead looks great’*, or spontaneous and opportunistic metonymical construals as in *‘The double cheeseburger wants another Coke’*. Some cognitive and psycholinguistic phenomena that support (indeed, appear to make possible) human imagination that have been well studied are conceptual framing (Fillmore, 1976, 1982), fictive motion (Talmy, 1996, 2000), conceptual metonymy (Lakoff & Johnson, 1980), conceptual metaphor (Lakoff & Johnson, 1980; Lakoff, 1982), and conceptual blending (Fauconnier & Turner, 2002; Fauconnier, 2018). The study of how these mechanisms play a fundamental role in the creation and development of many conceptual systems in mathematics can be found in Lakoff and Núñez (2000). Here, due to space limitations, we will only briefly describe conceptual framing, fictive motion and conceptual blending.

Frame semantics, the theoretical tool for studying conceptual framing, is an influential approach within cognitive linguistics, and in cognitive semantics in particular. Initially developed by Charles Fillmore (1976, 1982), it focuses on the continuity between natural language, its functions, and ways of bringing forth meaningful construals based on basic everyday experiences. This approach provides a method for studying the meanings of words in communicative contexts, and it is supported by results obtained in various areas of human cognition such as analogy, categorisation, and associative reasoning. In cognitive semantics, a frame is ‘any system of concepts related in such a way that to understand any one concept it is necessary to understand the entire system’ (Petruck, 1996, p. 373). For example, the concept of Buyer^3^ (and the word with which it is designated) acquires meaning within a conceptual network where there is also an agent—the Seller—that sells some good, where the good has some monetary value—the Price—for which the seller receives money when transferring the good to the buyer, and so on. Fillmore argues that to understand the meaning of each of these terms it is necessary to operate with a conceptual “frame” that relates the parts in a systematic way. In this example, this would be accomplished by the Commercial Transaction Frame that via a specific semantic network establishes the meaning of its elements and the relationships between them (Fillmore & Atkins, 1992). Although this frame is generic, it can be adapted and extended—*parameterised*—to particular and specialised cases such as the purchase and sale of intangible services, health insurance, etc. Similarly, in geometry, the concept of Hypotenuse, for example, can only be understood within a system of conceptual relationships that include the concept of Right Angle and Leg (Langacker, 1991). Without these, the concept of hypotenuse cannot be conceived of, since it acquires meaning in conjunction with the other elements of the frame: the Right-Angled Triangle Frame. In our argument, the concept of frame is crucial for understanding the cognitive semantic elements underlying the various conceptions of points at infinity in projective geometry that appear in the historical record as well as in current mathematical reference texts and textbooks.

Fictive motion is an important cognitive phenomenon supporting abstraction that is used throughout our argument. This cognitive mechanism is manifested linguistically in everyday expressions such as *‘The fence runs through the forest’*, or *‘The Equator passes through many countries’*, in which physically real static entities (a fence), or imaginary ones (the Equator), are treated as if they were dynamic. This phenomenon was first studied in detail by cognitive linguist Talmy (1988, 1996), who argued that fictive motion does not only pertain to the domain of words but that it actually reflects a process of conceptualisation in terms of motion. According to this theory, fictive motion is a cognitive phenomenon through which we often unconsciously (and effortlessly) conceptualise static entities in dynamic terms, as when we say *‘The tunnel goes through the mountain’*. Talmy analysed many linguistic expressions taken from everyday language in which static scenes are described and thought of in dynamic terms. Further empirical work has found experimental evidence of the psychological (Matlock, 2004, 2006), gestural (Núñez, 2006), and neural (Saygin et al., 2010) reality of fictive motion. Not surprisingly, fictive motion is routinely recruited in oral and written treatments of limits and continuity in calculus as manifested in expressions such as *‘f(x) approaches the limit L, as x tends to infinity’* (for details, see (Lakoff & Núñez, 2000; Núñez, 2006; Marghetis & Núñez, 2013)).

Another important building block for abstraction that is central to our argument is conceptual blending, a cognitive phenomenon that has been primarily studied by Gilles Fauconnier in collaboration with Mark Turner and others (Fauconnier & Turner, 2002). The following example, provided by Fauconnier and Turner, gives a sense of the basic principles of conceptual blending. The authors noted a commentary that involved boat sailing that read:

‘The clipper ship Northern Light sailed in 1853 from San Francisco to Boston in 76 days, 8 hours. That time was still the fastest on record in 1993, when a modern catamaran, Great American II, set out on the same course. A few days before the catamaran reached Boston, observers were able to say: At this point, Great American II is 4.5 days ahead of Northern Light’ (2002, p. 63).

From a cognitive linguistic perspective, what must be explained in this description is how the catamaran Great American II be can be “ahead” of the clipper ship Northern Light if the two boats sailed the route 140 years apart? Fauconnier and Turner point out that the meaning of “the catamaran being ahead of the clipper” can only make sense if it is construed as a single event; as an imaginary race that enacts a scenario in which the two boats are actually competing. According to conceptual blending theory, this scenario occurs in a mental space called “blended space” that has an internal logic and inferential organisation. That is, the scenario is brought forth in a purely imaginary conceptual domain that “blends” two (or sometimes more) input spaces according to certain cognitive principles. In this example, the meaningful construal blends the two events—the clipper sailing fast in 1853 (Input space 1) and the catamaran sailing fast in 1993 (Input space 2)—into a single event in which the “race” takes place. Similarly, Fauconnier and Turner have argued that conceptual blending occurs in the conceptualisation and treatment of many counterfactuals, such as conditional statements of impossible scenarios expressed in sentences like *‘Had you been a dog you wouldn’t have trusted that food’*. In this case, a blended space is put together in which certain (but not all) selected features of dogs are present (like having an excellent sense of smell), while keeping the human-like properties of the addressee “you”. A particularly interesting and powerful case of a counterfactual is that of actual infinity—a concept that while invoking endless processes have nonetheless final resultant states. This feature, it turns out, is central to the concept of point at infinity in projective geometry.

### Cognitive semantics of infinity

**Fig. 1:**
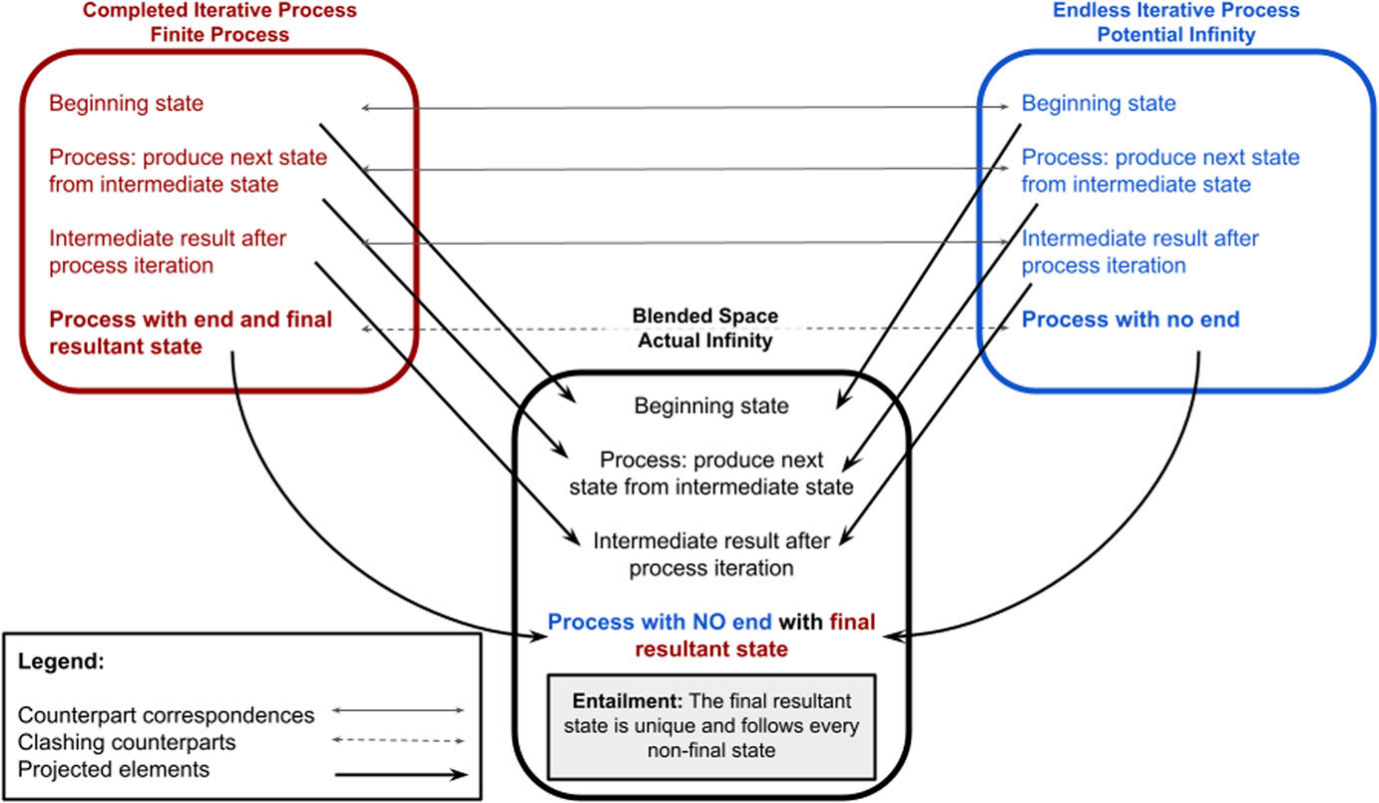
The BMI, the Basic Mapping of Infinity, as a double-scope conceptual blend. Adapted from (Núñez, 2005)

Infinity has presented a challenge for millennia. Potential infinity, characterised as an unending process, is contrasted with actual infinity, a completed, realised entity, with a final state. How can we consistently and stably conceive of a forever continuing process, with a final state after which there is no other? Núñez (2005) argues that the stable inferential system of actual infinity, is the result of a double-scope blend, with two input spaces (Fig. 1), denoted the *Basic Mapping of Infinity* (BMI). The first input space is that of Completed Iterative Processes. The second input space is that of Endless Iterative Processes.^4^ These input spaces correspond highly with one another, but in the former the process ends and has a final state, while in the latter the process has no end. In the blended space, these two elements are composed to yield a *process with no end, with a final resultant state*. This is a new inferential structure,^5^ not previously available in either of the input spaces alone.

**Table 1:** Directed line segment frame

Element	Description
*AB*	A line segment with a fixed start point *A*, and a fixed endpoint *B*
*D*	The length of the line segment *AB*

**Fig. 2:**
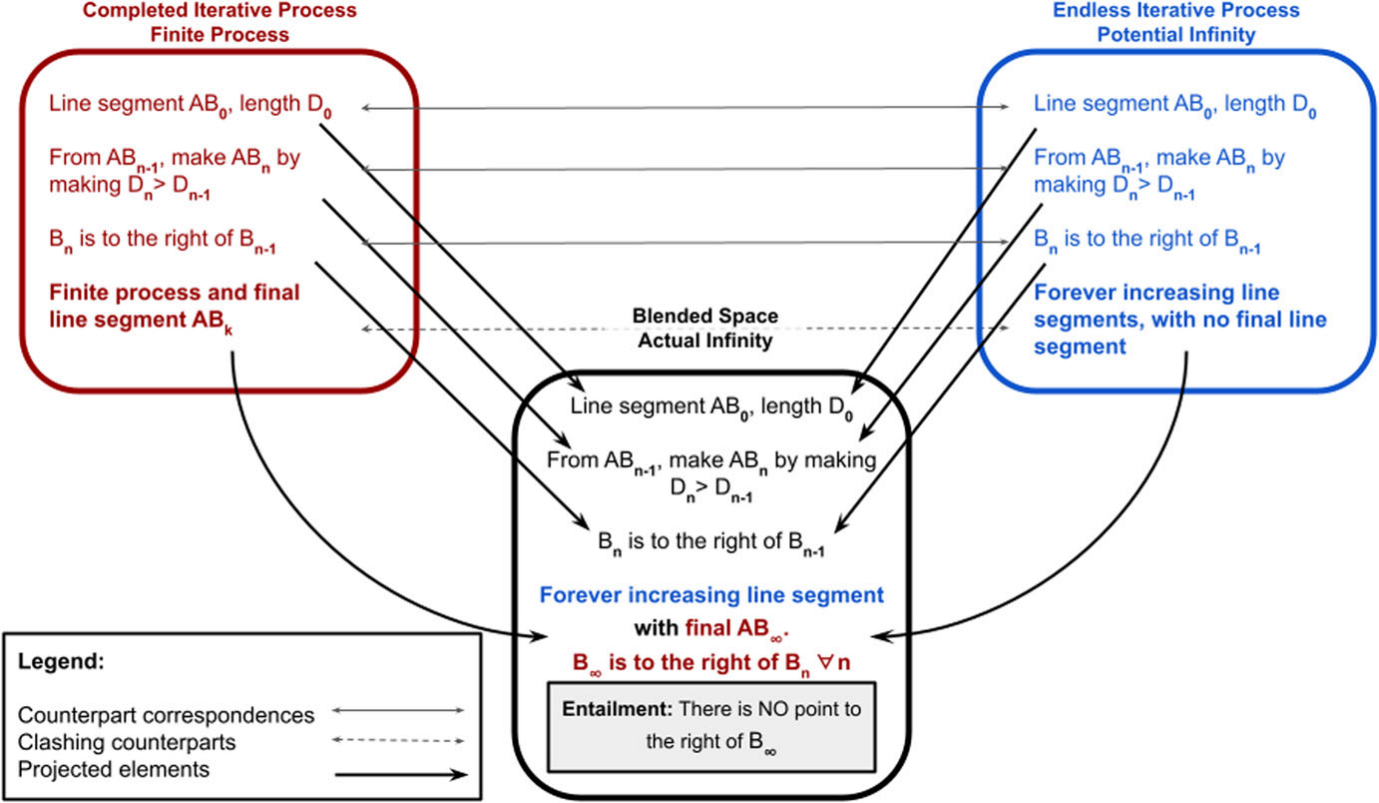
The BMI parameterised by the Directed Line Segment Frame

To apply this general cognitive mechanism to specific cases in mathematics one must *parameterise* the generic mapping. The first input space must be a *specific* completed iterative process, and the second input space must be an endless iteration of the same *specific* process. As an example, consider the Directed Line Segment Frame (Table 1), and the process of iteratively increasing the length of the line segment, for instance, extending it to the right. Parameterising the BMI via the Directed Line Segment Frame, as shown in Fig. 2, has the first input space be a finite number of iterations of extending the length *D* of the line *AB*, yielding a final directed line segment, $AB_k$, and has the second input space be an endless iteration of successively longer directed line segments $AB_n$ with no final, completed, directed line segment. By operating with the BMI under this parameterisation, we yield a blended space with an endless sequence of successively longer directed line segments *but* with a final resultant directed line segment $AB_{\infty }$, and the important entailment that there is no point on the line further than the final endpoint $B_{\infty }$. This resultant state has a point at infinity. In this way, there can be consistency and stability of meaning and inference when considering a point at infinity grounded in our domain general cognitive mechanisms.^6^ We are not here subscribing to a cognitive determinist account of mathematics.^7^ Rather, within a conceptual frame or blended space certain inferences are entailed by the structure of the frame or blended space.

## Projective geometry

Geometry, as a theoretical mathematical discipline, was first consolidated by Euclid circa 300BC. The axioms and inference rules in his *Elements* remained largely unchallenged and unchanged for the following 2000 years. During the Renaissance, the method of perspective drawing developed, in which parallel lines were drawn so as to appear non-parallel in order to convey depth. These lines were perceived (and conceived) as converging to a point on an imaginary horizon. Through this new method, developed outside of mathematics, the idea that parallel lines could meet—an idea forbidden in Euclidean geometry—was born. Under the requirement that parallel lines meet, it must then be answered where they meet. This meeting cannot be at any finite point, for then the lines would cease to appear parallel, thus the lines must intersect at a non-finite point, namely a *point at infinity*.

### Central cognitive conflict

Infinity, and the cognitive semantics and paradoxes associated with its introduction, have been examined, and accounts have been offered grounding their cognitive semantics in (Lakoff & Núñez, 2000; Núñez, 2005) as mentioned above. Here instead we focus on a separate **cognitive conflict** related to the introduction of this point at infinity, namely:

It appears that each line should have two points at infinity, one at each end of the line, but only one such point is canonically introduced.

This conflict arises out of this specific opposition: the apparent existence of two points at infinity, and the requirement that there be only one. These two elements do not emerge from a vacuum.

The **first element** in this opposition, the apparent existence of two points at infinity, is the entailment of the conceptual frames within which mathematicians conduct inference. As we shall see in Sect. 3.3, 3.4, and 3.5, the application of fictive motion or the reliance on the BMI paramterised by the Directed Line Segment Frame, yields the inference that at the end of the line there should be a point at infinity. This inference is valid whether the motion of a point, or the extension of a line segment, is leftward or rightward. In order to accord with mathematician’s intuitions, at either end of the line there should be a point at infinity. The **second element**, the requirement that there be only one point at infinity, results from a desire to preserve some inferences of Euclidean geometry and to remove exceptions (as we shall see in Sect. 3.3). In Euclidean geometry every pair of points defines exactly one line. If each line had two points at infinity, shared with all of its parallel lines, then these two points would define a family of lines rather than a specific line. Similarly, if parallel lines share only one point (the single point at infinity on each line), then one can state that every pair of lines intersects at a single point, and thus remove the exception for parallel lines.

However, as we will aim to demonstrate and support in the remainder of this paper, these two elements are only in opposition in the context of a critical **third element**: the global unitary mode of inference. The intuition that there is a point at infinity at the end of the line, and that this is true in both directions, only conflicts with the requirement that there be only one point at infinity when inference is required to occur in the global unitary mode. This is most easily understood through contrast with the situation in the local ad hoc mode, which is developed throughout the following sections but which we presage here. A local ad hoc inferential structure allows for a patchwork of isolated, conceptual frames within each of which inference is performed. In this case-study, these conceptual frames correspond to leftward and rightward extensions of line segments (or motions of points). Within each of these conceptual frames there is exactly one point at infinity. This point at infinity is constructed, and in a modern sense defined, on the basis of the elements of the frame and in response to specific inferential needs. It is in this sense we call this mode ad hoc^8^ in that it is ‘formed or used for specific or immediate problems’ and ‘fashioned from whatever is immediately available’ (Merriam-Webster, n.d.). The ad hoc construction of the point at infinity within these conceptual frames renders them ineffable in the other frame—for instance, one cannot consider (within the terms of the conceptual frame) the *left-endpoint at infinity* of a line segment that is extended rightward—and thus the points in these separate frames cannot be substituted for one another. It is in this sense that we say these frames are local, in that they are inferentially isolated from one another; from within, there is no means to consider the difference, equivalence, or co-existence of these two points at infinity. In any coherent inferential problem within this local ad hoc mode, there can be only one meaningful point at infinity. Thus, this inferential structure as a whole is not in conflict with the requirement, should it be raised, that there is only one point at infinity. When checking if the constraint is satisfied any check occurs in an isolated frame, checks in all isolated frames agree, and there is no super-ordinate frame or element of the inferential structure in which a clash might be detected. However, when inference is performed within the global unitary mode these two points at infinity are meaningful within a single frame, or the separate frames are required to be reconciled into a coherent whole,^9^ allowing for substitutability and thus questions of equivalence, difference, co-existence etc. This then gives rise to the cognitive conflict, yielding questions like:

How can the seeming two points at infinity be one? They appear to be infinitely far apart. One is to the left of every point the other is to the right of every point.

In sum, in this specific case-study the cognitive conflict arises out of this threeway tension between:

1. the entailments of the directed conceptual frames invoked by mathematicians,
2. the requirement that there be only one point at infinity,
3. and the metamathematical constraint that inference be performed in a global unitary mode.

It is only in the presence of such an opposition that the cognitive conflict can arise.

How then, do mathematicians stably and consistently conduct inference in a system that appears to have such a glaring cognitive conflict at its centre? A conflict which seems to introduce barriers to consistent inference. How are these barriers overcome? Do mathematicians elide or resolve the conflict? Is the mode of inference intentionally or implicitly altered in response to the cognitive conflict? If so, do we see these choices throughout the history of mathematics? Modern mathematics has a host of solutions, to which we will attend shortly. With respect to the questions at the heart of this paper, we consider these modern solutions and practices in comparison with their historic predecessors to answer several questions:

Are the modern constructions implicit in the earlier work? Do the different stages of development share a ‘common thread’? Do changes arise from ‘hidden tensions’ and endogenous issues?

To address these questions we analyse three distinct textual sources: modern mathematical reference texts Sect. 3.2, modern mathematical textbooks Sect. 3.3, and seminal texts in projective geometry (Sects. 3.4 and 3.5). The reference texts serve as a normative, sanctioned source of mathematicians’ shared inferential systems. We draw the common constructions of inferential systems for projective geometry from Bourbaki (1998), Gowers et al. (2008), and nLab (2022a, b). We analyse their structure with respect to the formal tools they invoke for performing inference concerning the point at infinity, which we can use as a standard for comparison to that of the textbooks and seminal texts. However, the practice of mathematics often differs from the sanctioned, normative systems that are brought to bear in formal inference. We analyse modern textbooks to gain an insight into the inferential practices invoked during mathematical problem solving, as opposed to the sedimented formal systems of the reference texts. The textbooks we analyse are Veblen and Young (1916), Coxeter (1961), and Courant and Robbins (1996). On the basis of the analyses of these two sets of texts we argue that there are two conceptually incompatible inferential structures for projective geometry. We then analyse the seminal works of Desargues (1639) and Poncelet (1865), develop an account of their approaches to inference concerning this cognitive conflict, and argue that the pre-modern inception of projective geometry is conceptually incompatible with the modern normative account. In light of these findings, grounded in cognitive linguistic analyses, we claim that philosophies of mathematics that rely on historical accounts of progress in support of mathematical realism must be adjusted, abandoned, or restricted to accommodate results of this nature.

### Reference texts

Within these reference texts, there are two primary patterns of organisation present in constructions of the real projective plane. We will here refer to these general patterns as *approaches*, to distinguish them from the particular constructions. These approaches we will refer to as the *gluing approach* and the *stipulative approach*. The *gluing approach* relies on the formal tools of equivalence classes, identifications, and quotient spaces to provide a method for dealing with separate objects or separate representations of objects as the same with respect to formal, inferential practices. In contrast, the *stipulative approach* relies on altering the semantics of ‘point’ and ‘line’ in the object-language. These approaches are embedded within the modern mathematical context, operating in a global, unitary, inferential mode, and thus in a context in which the cognitive conflict is present.

In both cases the cognitive conflict is resolved by removing its relevance for conducting inference. Inference is marshalled by formal methods which are constructed such that they satisfy the requirements and are relatable to the corresponding conceptual frames. This approach does not remove the requirement, nor does it deny the intuition of the conceptual frame, it resolves the cognitive conflict by providing a method for conducting stable inference—perhaps leaving metaphysical questions intact, but in a manner such that these questions do not affect the inferential practice. If viewed in isolation one might consider that the cognitive conflict is elided by these *approaches* because the conflict cannot arise within them. However, the cognitive conflict only arises as a result of the opposition between metamathematical requirements and the conceptual frames of the mathematicians; these approaches deny neither of these constitutive components and thus do not elide the conflict, rather they leave the cognitive conflict intact but render it inferentially irrelevant through the construction of appropriate formal methods that satisfy the constitutive elements of the cognitive conflict in a fashion that allows for stable, consistent inference.

To make these features plain we detail two particular constructions exemplifying these approaches. The *gluing approach* is exemplified by the *planar construction* which takes the extended Euclidean plane—the Euclidean plane in which each line has two points at infinity (represented by the symbols $+ \infty$ and $- \infty$)—and identifying these two points at infinity as equivalent.^10^ Formally, equivalence is characterised by a relation which respects the formal deduction rules, this is what Gowers et al. describes as ‘[what it means] to “regard two mathematical objects as equal” when they are not equal?’ (2008, p. 25). This means that wherever $+\infty$ appears in inference it can be replaced by $-\infty$ without affecting the validity of the inferences, similar in effect to the way that 3 can be replaced by $\log _{10}(1000)$ in any arithmetic statement. Thus, the effect of the cognitive conflict in practice is formally resolved, it cannot affect inference.^11^

The *stipulative approach* is exemplified by the *synthetic construction* as evidenced in Gowers et al.:

‘A fourth way to define the projective plane is to start with the usual Euclidean plane and to add one “point at infinity” for each possible slope that a line can have’ (2008, p. 267).

Each line contains one such “point at infinity”.^12^ These “points” were not initially members of the Euclidean plane, they are formal primitives which are added in a particular manner and satisfying particular axioms. Creating this inferential structure relies on altering the natural semantics of point such that a ‘point’ is only something that satisfies certain properties, and similarly for ‘line’, rather than the natural semantics of the conceptual frame wherein a point is a position or location or a marker of such. Even in this terse reference text we see an acknowledgement of this altered semantics by the use of quotation marks concerning this element at infinity, although in formal mathematics this “point at infinity” is no different from any other projective point. A similar approach, and typographical acknowledgement of difference, is presented in nLab (2022a): ‘we can construct the projective plane $[\mathbb {R}]P^2$... [b]y starting with $[\mathbb {R}]^2$ and adding a “point at infinity”. These primitives are introduced with a relationship to parallel lines, but these points are not locations in a conceptual frame. Sanctioned inference then can proceed syntactically. Though there is no prohibition against using conceptual frames for intuition, a point at infinity in such a frame (e.g. constructed via the BMI) is not equivalent inferentially to the synthetic point at infinity.^13^ These frame-based points and inferences might cause practicing mathematicians to make inferences, but they do not warrant these inferences. Thus the effect of the cognitive conflict in practice is resolved formally, as within the object-language there is no second point at infinity of which to speak so the cognitive conflict cannot affect inference, as the only warranted inferences are those rendered into this *stipulative approach*.

Both the *gluing* and *stipulative* approach yield a formal manner of conducting inference with respect to the point at infinity, that satisfies the needs of projective geometry operating in the global, unitary mode. To be specific, this means that there is a single, coherent definition/construction of the point at infinity, and that inferences concerning the point at infinity are independent of the context in which they arise. The warranted inferences are those that can be articulated within these formal constructions. Within these formal constructions, these object-languages, there is no possibility of clashing inferential results. Any representation of the point at infinity, or different points at infinity, are inferentially equivalent. They can be substituted for one another at any stage in inference. The formal constructions stabilise inference especially when dealing with the point at infinity. With respect to inference, the cognitive conflict is resolved by these constructions and the approaches in general.

### Textbooks

Mathematics, even mathematical texts, are much richer than the simple statement of the formal characterisations of domains and objects within the reference texts above. Textbooks, generally intended to be accompanied by instruction, are designed to educate the reader not only in the facts of mathematical knowledge but also in the mode of constructing and discussing mathematics. Analysing mathematical textbooks provides insight not just into the result of the developmental trajectory of the subject but also into the sanctioned practice of how one should work with the sanctioned result. Within our selected standard textbooks for analysis, we analyse specifically their introduction of the point at infinity, and the manner in which they use the point at infinity. We separate the discussion into these two sections as the manner of introduction is often normative and explicit, while the manner in which the point at infinity is used often implicitly contains much more than is present in the definitions, thus providing differing insights.

#### The introduction of the point at infinity

Authors of all three of the textbooks we surveyed give clear introductions of the point at infinity. For each author the initial motivation to introduce a point at infinity is to remove exceptions

‘it would clearly add much ...if we could regard them [Euclidean axioms] both as true without exception. This can be accomplished by *attributing to two parallel lines a point of intersection*’ (Veblen & Young, 1916, p. 7, emphasis in original).

Each author takes a *stipulative approach* to this introduction. Veblen and Young and Courant and Robbins both define the point at infinity synthetically. They *‘attribute to two parallel lines a point of intersection’* which they call the point at infinity, with the properties required such that the exceptions are removed. Coxeter reasons that a point is determined by a pencil of lines, including a pencil of parallel lines, and takes these pencils to define the projective ‘points’. In all of these cases we can plainly see the *stipulative* nature of their approach. The meaning of the point at infinity is either defined by fiat and constrained to satisfy certain desired axioms, or the meaning of point is altered metonymically to mean a set of lines: ‘[the] point at infinity $A'$ is really only another name for the pencil of lines parallel to p’ (Coxeter, 1961, p. 5).

With respect to the naming convention for the ‘point at infinity’, only Courant and Robbins go so far as to give an explicit motivation for such a naming, stating

‘if a straight line that intersects another is rotated slowly towards a parallel position, then the point of intersection of the two lines will recede to infinity’ (1996, p. 180).

While their definition is *stipulative*, their explanation of the naming convention relies on a framework beyond that of their synthetic approach. In this text we can see they clearly rely on fictive motion, specifically line-rotation and point-locomotion.^14^ This implicitly introduces into the inferential practice a non-synthetic semantics for the point at infinity, requiring points to be locations within a conceptual frame in which inference is performed. Further, they only rotate ‘towards a parallel position’. By only considering rotation towards this position, they invoke a frame in which the point can only move in one direction, say rightward. This rightward motion is considered as completed, via a parameterisation of the BMI, with the final end state being to the right of every previous location and beyond which there is no other. Thus the query as to whether this point is also at infinity but on the left hand side is meaningless within that frame, the terms of the query are ineffable within that frame. Without considering rotation beyond the parallel, the issue of the point moving from the right hand end to the left hand end is not frame-relevant. In their specific invocation of conceptual frames with location, inference, or in this case motivation of definitions, occurs in a local mode, rather than a global one. However, Courant and Robbins go further

‘The intuitive concept of a point on a line receding to infinity might suggest that we add two ideal points to each line, one for each direction along the line. The reason for adding only one...[is] to preserve the law that through any two points one and only one line may be drawn.’ (1996, p. 182)

They note that one might consider adding two separate points at infinity to each line due to the fact that there are two possible frames (as they have constructed them), one for each direction, each appearing to require a point at infinity. They stipulate that only one such point be added, without an explanation as to how one should consider—and, importantly, treat in inference—the two points at infinity in these separate frames as the same, or equivalent, in the global unitary mode required in modern mathematics. In so doing, they explicitly raise the constitutive components of the cognitive conflict.

**Fig. 3:**
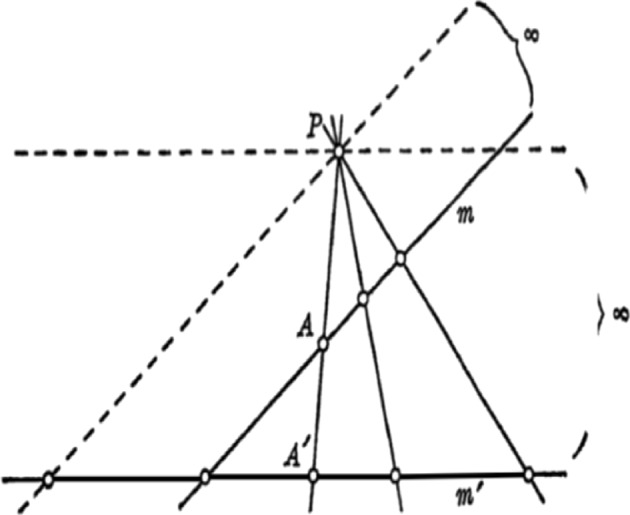
One-sided infinity in Veblen and Young (1916, p. 13)

**Fig. 4:**
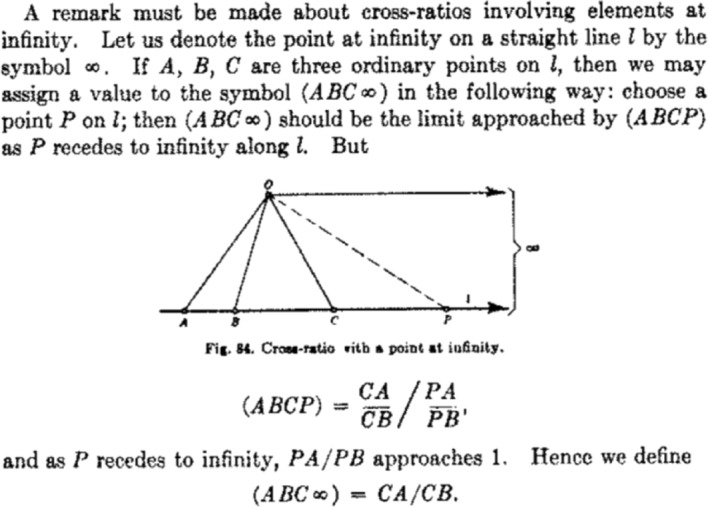
One-sided infinity in Courant and Robbins (1996, p. 185, reproduced with permission)

#### The use of the point at infinity in inference

The authors acknowledge the intuition that there should be two points at infinity on each line but instruct that there be only one, thus dismissing this cognitive conflict without resolution, simply leaving the terms of the conflict standing in opposition. However, their use of the point at infinity implicitly educates the reader in the manner of dealing with this cognitive conflict. In Fig. 3, reproduced from Veblen and Young, and in Fig. 4, reproduced from Courant and Robbins, we see that despite their statement that there is only one point at infinity, and that by our ‘intuitive concepts’ it must appear to us on both ends of an infinite line, when representing it pictorially they only do so on one side of the line in any individual figure. In Veblen and Young, the language used is explicitly static,^15^ referring to correspondences between points of lines. It is only in attending to the accompanying diagram that an apprentice mathematician is instructed that the point at infinity is to be located at one side. The authors could easily have represented the infinity symbol symmetrically on both sides of the pairs of parallel lines, but they did not. In the case of Courant and Robbins they do so explicitly invoking motion in the text accompanying the figure, referring to the point *P* as *receding to infinity*; and in the figure by the use of arrows, and by using *O* as a base point from which the line segment begins, in addition to representing the infinity symbol solely on one side.

If the point at infinity is merely a formal symbol or is to be treated in practice as a primitive, then why represent it anywhere, and why choose one side over another? In order to perform inference in practice, the location of the point at infinity is relevant if inference is performed within a frame invoking directions and motion. In dynamic frames the point is not just a mathematical primitive, but requires a location with respect to the other elements of the frame due to its construction via the BMI. In these examples this asymmetric representation of the point at infinity is not atheoretic or inferentially neutral, it instructs the apprentice mathematician that in any inference they need only consider the point at infinity as on one side of the pair of parallel lines. Why does treating the point at infinity as if located on one side succeed in producing stable inference when it is clearly at odds with the constraints that the authors explicitly note. The authors provide no explicit warrant as to why this approach can be taken. However, when considering the cognitive semantics of frames invoking motion we can find an answer. Considering Courant and Robbins specifically, rotation towards the parallel *but not beyond*, ensures that the point *P* only moves rightward toward infinity. Prior to the line being parallel the point *P* is always to the right of, for example, the point *C*. Invoking fictive motion within such a frame yields the inference that when this rotation is complete (i.e. when all angles less than $90^{\circ }$ have been progressively exhausted), the final resultant state of *P* is to the right of every point, as depicted. Outside of such a conceptual frame the question of whether *P* is infinitely left or right would give pause for thought, but within this conceptual frame the cognitive conflict cannot arise. In order for the conflict to arise, there must necessarily be an opposition of elements. However, one of the necessary components of the tension—the global unitary mode of inference, within which the point at infinity on the left and on the right are both meaningful—has been implicitly suppressed and thus the cognitive conflict elided. Although Veblen and Young do not invoke motion in the text, or in their diagrams, their representation of the point at infinity only on one side also instructs the reader that only one of the two possible locations need be considered within any conceptually cohesive problem; i.e. within any one frame only one of the two possible locations is relevant to inference.

A skeptic might argue that the representation of the point at infinity on the diagram, the arrow heads, the rotation to but not through the parallel, is all mere window dressing. This raises the following question: is the choice to represent infinity on only one side of a line at one time merely to simplify discussion, or does this have a functional role in the construction of mathematics, at least in this pedagogical setting? For this we look to Fig. 4 again as a case-study.

#### Case-study: defining the cross-ratio

The cross-ratio is a geometric construction pertaining to any collection of four co-linear points. As we can see in Fig. 4, the cross ratio of four finite points is defined as

$$\begin{aligned} (ABCP) = {\frac{CA}{CB}}\bigg /{\frac{PA}{PB}} \end{aligned}$$

The referenced line segments are directed (or signed) line segments, meaning that $CA = - AC$. The question presented is how to define (*ABCP*) when the point *P* is at infinity. Courant and Robbins explain that as the point *P* *recedes* to infinity, the ratio $\dfrac{PA}{PB}$ will *approach* 1. At this stage we already see the role of fictive motion in constructing this definition, but in fact the fictive motion is doing more than simply grounding the concept of limits. Since *PA* and *PB* are in the same direction, namely *P* is to the right of both *A* and *B*, both *PA* and *PB* have the same sign. Similarly, if *P* had instead moved leftward toward infinity, then *AP* and *BP* would also have the same sign. Thus, in either case, the limit of $\dfrac{PA}{PB} = +1 = \dfrac{AP}{BP}$. This is important, mathematically, as it shows the cross-ratio is well-defined: it doesn’t matter *how* *P* approaches infinity, the result will be the same.

However, the fact that both of the line segments connecting *A* and *B* to the point *P* have the same sign, the same order, the same direction, is an entailment of the fact that *P* *moved* to infinity. The definition may not rely on *how* *P* approaches infinity, but it does rely on the fact that *P* does in fact *move to* or *approach* infinity. As the movement entails that if *P* is to the left of *A* it is also to the left of *B*, and thus the signs of the line segments will be the same, and this remains the case once *P* reaches the point at infinity.

Suppose instead that the definition did not rely on fictive motion. The point *P* was instead just *at* the point at infinity, with no movement to reach that location. Then, in order to define the cross ratio, one must decide which direction, or sign, to apply to the line segments connecting *P* to *A* and *P* to *B*. If both directions are chosen to be the same, then their signs will be the same. If the directions are chosen to be different, then their signs will differ. In the former case, $(ABC\infty ) > 0$ and in the latter $(ABC\infty ) < 0$. In this we can see that fictive motion, and the directional entailments it provides, play a functional role in the construction of this definition. Without the dynamic frame there is a choice issue that requires resolution, but this choice is elided in the dynamic frame.^16^

One of the symptoms arising from the cognitive conflict—namely, the point *P* being at infinity on one side and the other, or there being two possible directions from the point *P* at infinity to some finite point—cannot be meaningful within the dynamic frames as constructed by Courant and Robbins. Fictive motion, with the parameterisation of the BMI, entails that the frames are always directed and only one side need be considered within any inferential context; in these directed local frames there is a single point at infinity, and so only one point at infinity is ever inferentially relevant. The construction of cross-ratio is equivalent for both directions, and is therefore well-defined in that it allows for stable inference. However, it does this through the agreement of a patchwork of isolated frames, rather than through a global unitary mode of inference. This is a feature of the local ad hoc invocation of the point at infinity within directed frames. There are two separate directed frames, each with a point at infinity constructed as the end of an infinite motion in that direction. Inference only occurs within one of these frames in any problem situation, and answers can be compared across these two frames to ensure consistency and thus allow for inference to proceed stably in all contexts—whether or not the practitioners are aware of this feature.

However, the requirements imposed by a global unitary mode are not met by this local ad hoc mode. In the global mode, even if directed frames are invoked, there is requirement that the inferences concerning the point at infinity are independent of the context, i.e. the specific isolated frame of the patchwork in which they arise. If there are multiple frames in which the point at infinity arises, these must all be reconciled together, either by constructing a new monolithic frame or via formal methods that inferentially stitch together these local frames such that they are no longer isolated from one another. Defining the cross-ratio in the *gluing approach* or *stipulative approach*—formal systems constructed in the global mode—necessitates the removal of signed distances (in the definition and in general) due to the choice issue mentioned previously and the requirement of substitutability. However, these concerns and resolutions are neither meaningful in, nor resolved by, the implicit application of local ad hoc mode here invoked. These issues, questions, and resolutions, are only made meaningful by the global mode, by regarding all frames in which a point at infinity arises as part of a global unitary context. The local ad hoc mode renders these issues and their resolutions meaningless as they are never frame-relevant, and there are no inferences within the object-language of projective geometry that are left wanting for a resolution, nor ‘hidden tensions’ or endogenous issues within the domain.

These two textbooks, Veblen and Young (1916) and Courant and Robbins (1996), elide the cognitive conflict in the context of performing inference. While there may be formal answers, through *gluing* or *stipulation*, these authors implicitly instruct apprentice mathematicians that in practice the cognitive conflict can be dealt with by elision through reliance on specific cognitive semantic frames—specifically, isolated frames invoking direction in which the point at infinity is constructed ad hoc and its inferential use is restricted to its frame of construction. Invoking directed frames in which motion can only proceed in one direction and only becomes farther and farther removed on that side, renders the cognitive conflict irrelevant in the performance of inference. Within that directed frame, there can be only one frame-relevant meaning for the location of the point at infinity or other related concepts. Thus, apprentice mathematicians are implicitly instructed that they need only conduct inference in such frames and thus elide the cognitive conflict in practice, for instance, by only approaching infinity but not *passing through infinity*, or equivalently by rotating toward the parallel but not *through the parallel*.

However, an inquisitive student or mathematician concerned with foundations or generalisation might wonder what happens when we rotate through the parallel? Why rely on this peculiar restriction? While there may no be questions within the object-language of projective geometry, it may be important to instruct apprentice mathematicians in the global unitary mode to this domain. Though neither Courant and Robbins nor Veblen and Young ever elaborate this possibility, we can here look to Coxeter for instruction.

#### Case-study: fictive motion in the global unitary mode

**Fig. 5:**
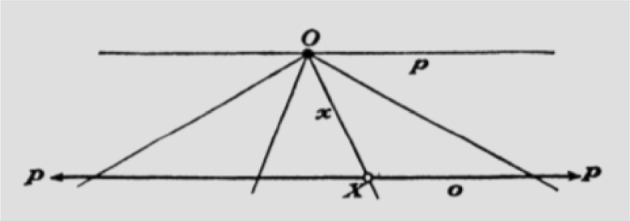
Two-sided infinity in Coxeter (1961, p. 21, reproduced with permission)

In Fig. 5, reproduced from Coxeter (1961), we see a stark contrast to the earlier figures. Coxeter is describing the correspondence between points on separate lines via a perspectivity, the same context as Veblen and Young. However, here motion is invoked through the linguistic expressions, as Coxeter states that ‘points on a line are said to form a *range*, especially when we regard them as the possible positions of a *variable* point X (*which runs along the line*)’ (1961, p. 21, italics in original, underlined italics added). *X* is clearly described in dynamic terms, running along the line. We also see arrowheads similar to those of Courant and Robbins, although they are present on both sides of the line. We quote here at length Coxeter’s description of the diagram:

‘The line *x* through *O*, rotating continuously, determines on *o* the point *X*, which runs along to the right, say, until *x* is parallel to *o*, then immediately reappears far away on the left and continues running to the right. In affine geometry the point *X* makes an infinite jump; but in projective geometry its motion, through the single point at infinity, is continuous’ (1961, p. 21).

Coxeter explicitly aims to deal with the full rotation of the line *x* through the parallel. In this again we see explicit reliance on fictive motion. Later in the text we again read ‘[w]e can reach the left side of the barrier point from the right by proceeding to the right and passing through the point at infinity’ (1961, p. 27). Recalling Coxeter’s *stipulative* definition of projective points as pencils of lines, it is not clear how a point *X* can pass through such a point, or even how motion is occurring at all. The reliance on fictive motion, the planar features of the diagram, and the double marking of *P* at either end of the line, encourage the reader towards a *gluing* approach when actually engaging in inference. This becomes even more evident later with the explicit textual statement that ‘$\infty ( = - \infty )$’ (1961, p. 33). The invocation of fictive motion requires locations at which *X* can be, recourse to formal points is not sufficient in order for this frame to be useful.

However, the story is richer than this move from explicit *stipulation* to implicit *gluing*. Coxeter does not simply just introduce rotation through the parallel and then all else proceeds as per Courant and Robbins or Veblen and Young. In allowing for rotation through the parallel he has maintained the global unitary mode of inference while invoking fictive motion, an important break from the previous authors—one can no longer treat the point at infinity in the local ad hoc fashion of Courant and Robbins, the cognitive conflict is not elided or suppressed. This introduces inferences and issues that were not previously false but rather were previously meaningless. The most obvious case is the inference that *X* can move through infinity. Statically, the claim that there are points arbitrarily close to $\infty$ on the right and the left is true in the inferential structures of Courant and Robbins and Coxeter, but only in Coxeter is motion through $\infty$ possible due to his global unitary mode of inference.

While this move to a global unitary mode within the context of fictive motion introduces new inferences, there are issues that arise from this shift too. For instance, if we attend carefully to the language used we see Coxeter does not mention when the point *x* is at the point at infinity. He refers to the point moving rightwards, and then *immediately reappearing* far away on the left. Although his language is initially that of naturally continuous motion, his discussion at this thorny moment becomes discontinuous. In order for something to *reappear* it must first have *disappeared*. How can something moving continuously achieve this? Further, at the moment that *x* is parallel to *o* the point is not ‘far away to the left’, at that moment it must in fact be at infinity. Having reached the point at infinity, the point *X* ‘continues to the right’. How does it make its first movement away from infinity? The completion of the move toward infinity is made cognitively meaningful through conceptual blending and the BMI, but no such feature grounds this question of first movement. It is an issue for mathematical practice in projective geometry only as a result of this move toward a global unitary mode. This fictive motion, in the context of a global unitary mode, results in new truth statements which were previously meaningless and new problems and questions that could not previously have been posed. These truths and problems are not in the object-language, but occur only in the extra-geometric language of the mathematical practice. These new statements do not arise due to inconsistencies or ‘hidden tensions’ that were ignored in the local ad hoc constructions of Courant and Robbins or Veblen and Young, but due to a metamathematical requirement that the constructions are independent of context, that the inferential operations adhere to the global unitary mode.

To summarise, in these textbooks the real projective plane and the point at infinity are introduced normatively via the *stipulative approach*. In practice, the point at infinity is invoked in dynamic cognitive semantic frames. This introduces the requirement that the *stipulative* definition be implicitly augmented such that the point at infinity has location rather than being a synthetic mathematical object. In this context, in the opposition between the normative requirement that there be a single point at infinity, the geometric intuitions grounded in conceptual frames with locations, operating in the global unitary mode of inference, the cognitive conflict arises. This cognitive conflict can be handled, such that stable inference can be conducted, in at least two ways. The first solution is to elide the cognitive conflict in the performance of inference. This is accomplished by invoking a patchwork of isolated frames in each of which there is only one frame-relevant construction of the point at infinity in any inferentially cohesive situation. Within any such conceptual frame there is no frame-relevant sense in which an element could be doubly-located or concern as to how a point moves from one of the points at infinity to the other. The cognitive conflict is elided with respect to the practice of mathematics, is not inferentially relevant, due to the structure of the frame and the local *ad hoc* construction of the point at infinity via the BMI within each frame. However, one should not understand this to imply that the use of local ad hoc frames *necessarily* elides the cognitive conflict. It is only in this pedagogical context in which the cognitive conflict is raised—a context with hybrid pedagogical goals, that attempts to scaffold effective inference while also developing an appreciation of the modern mathematical project—that this can be considered an elision. The conflict is raised as an issue, owing to the tension in modern mathematics between metamathematical requirements and the operation of conceptual frames. However, it is elided in practice in these texts, as there are stable and consistent means for performing inference via the local ad hoc mode which can develop apprentice mathematicians inferential skills without a need to dwell on the conflict or its resolutions, and this can be achieved without introducing inconsistencies. This elision is at odds with the formal characterisations of the projective plane which are explicitly global unitary.

The alternative to this local mode, while still satisfying the dynamic construal’s need for locations, is to invoke a global formulation evidenced above through the *gluing approach*. By conducting inference in the global unitary mode with frames invoking locations, while maintaining the requirement that there be only one point at infinity, the cognitive conflict cannot be elided. In this global unitary mode it is frame-relevant to question whether the point at infinity is on one side, or the other, or both. In order to apprentice mathematicians in the manner of producing stable inference within this global mode, the cognitive conflict must be resolved directly; the *gluing approach* is one such attempt. Further, operating in this global unitary mode does more than introduce inferences not meaningful in the local mode, but also serves to introduce issues requiring resolution which were not present in the local mode. These issues and inferences are not resolutions to issues within the local ad hoc mode. Rather they are introduced through the imposition of metamathematical constraints and mathematical constructions that satisfy values external to projective geometry and its theorems.

### Girard Desargues (1591–1661)

#### The introduction of the point at infinity

In Desargues’ *Brouillon project d’une atteinte aux événemens des rencontres du cone avec un plan* (1639) comes the first explicit introduction and use of a point at infinity in the historical record (Field & Gray, 1987). The introduction comes without any theatrics. Having introduced the term *‘ordonnance’*, ordinance, to refer to a set of lines all converging in one point which he calls *‘le but’*, the butt or the goal, he then states

‘Pour donner à entendre l’espece de positions d’entre plusieurs droites en laquelles elles sont toutes paralelles entre elles, il est ici dit, que toutes ces droites sont entre elles d’une même ordonnance, dont le but est à distance infinie en chacune elles d’une part & d’autre.’ (Desargues, 1639, p. 1)

*‘To convey the kind of positions in which several lines are parallel to one another, it is here said, that all these lines are of the same ordinance, whose target is at infinite distance on each of them on one side and the other.’* (Our translation)

In this Desargues’ introduces the concept that a geometric element, *le but*, can be at an infinite distance. He introduces this point in the singular, but requires that it be at an infinite distance on one side and the other. Perhaps one could interpret this as indicating a naive appreciation of the global unitary *gluing* approaches in which the point at infinity has two locations or the two infinite endpoints are treated as equivalent; ostensibly, this supports the claim that our modern understanding is implicit in this pre-modern work. In contrast, we will demonstrate that this is not the case by analysing Desargues’ use of the point at infinity for inference.

First, it bears noting that this is the full extent of the explanation provided as to the meaning of a point being at infinity. While this manuscript was written for contemporaries, with many of whom he would have had discussions, it is still striking that there is no further description as to the meaning of this drastically new point—this point that breaks from 2000 years of tradition. As if it were obvious that a reasonably trained mathematician of the time would enact the same meaning of this new concept without explicit instruction. Considering the scale of this conceptual leap, it appears that either the leap must have been heavily scaffolded by the inferential practices of the time (the frames invoked, methods deployed etc.), or that the cognitive conflict with which we are concerned is somehow not presented or salient in Desargues’ work or conceptualisation.

#### The use of the point at infinity in inference

Looking to the Desargues’ use of the point at infinity, we notice a peculiar pattern. When considering points on a line, and the distances between them, Desargues’ uses a host of arboreal terms: ‘a plethora of botanical names for simple configurations of points and lines which, taken together, only serve to obscure the text. Of those terms, we need only say that those which connote line segments... may be taken to mean ‘line’ or ’line segment’, whereas those which suggest points... mean ‘point’.’ (Field & Gray, 1987, p. 47) However, while these terms do serve to confuse the modern reader, they also invoke a Directed Line Segment Frame, in that ‘branches’ have a natural start at the ‘knot’ of the tree, and similarly ‘stumps’ are the origins of ‘trunks’. This use of terminology does not merely mean ‘line segment’ or ‘point’, but invokes a richer, directed framework.

The use of arboreal terms invoking directed line segments in the absence of other evidence would not be of interest, however it appears to be deployed in a rich network of directed frames which gives meaning to Desargues’ point at infinity. As an example, when discussing pairs of line segments that share a particular ratio Desargues considers what happens if one of these line segments became completely diminished:

‘Voila comme en un arbre la souche & le tronc depuis la même souche jusque à l’infini **d’une ou d’autre part d’elle**, y sont entre eux couple de branches extrêmes, dont la petite est à petissée jusque à la souche & la grande est alongée à l’infini’ (Desargues, 1639, p. 6, emphasis added)

*‘Thus in a tree, the stump and the trunk, from the same stump to infinity on* **one or the other side** *, are a pair of extreme branches, the smaller shrunk to the stump and the larger extended to infinity.’* (Our translation, emphasis added)

First, note that the ‘points’ do not move to infinity as detached from any other geometric element. It is only in the process of extending a line segment, grounded in the BMI parameterised by the Directed Line Segment Frame that the point at infinity is invoked. This is supported further by Desargues’ initial introduction of lines as

‘toute ligne droite est entendue alongée **au besoin** à l’infini d’une part et d’autre’ (Desargues, 1639, p. 1, emphasis added)

*‘every straight line is understood to be extended* **as necessary** *to infinity on one side and the other’* (Our translation, emphasis added)

in which infinitely long lines are not ontologically prior to the geometric work, but are constructed ‘as necessary’, ad hoc, via the BMI. Moreover, although Desargues initially introduces the target of parallel lines at infinity on ‘one side *and* the other’, he now states that the trunk is from the stump to infinity ‘on one *or* the other side’. This is a direct reflection of the BMI as parameterised by the Directed Line Segment Frame. *Prima facie*, the point at infinity is on both sides of every line. However, invoking points only as the endpoints of directed lines entails that a point at infinity could only have one frame-relevant location, at one particular endpoint, in any single conceptually cohesive problem—as a line can only be extended from a fixed base point in one direction at any one time. To ask whether the endpoint of this line, the point at infinity, is on both the left side and the right side is not meaningful in the context of extending line segments. This is very much in parallel with the isolated frames implicitly invoked in the local ad hoc mode of Courant and Robbins and Veblen and Young.

Taken together, the introduction and use of the point at infinity by Desargues’ goes some way to illuminating how stable, inferential systems can exist even when they do not accord with our formal characterisations as described in the modern reference texts. There is no globally applicable definition of the point at infinity, it is not a conceptual primitive of the domain. Moreover, we do not see naive versions of *gluing* or formal *stipulative* approaches in Desargues’ work. In relying on Directed Line Segment Frames, the point at infinity on the right could not be used in a local inferential frame concerned with lines extending to the left as it would have no meaning in that frame. Although the opportunity for confronting this cognitive conflict is ostensibly presented in the work, the grounding of Desargues’ geometry in Directed Line Segment Frames renders ineffable the question of whether the two distinct points at infinity are the same or different, and without the presence of modern metamathematical requirements for a global unitary mode of inference a necessary element of the cognitive conflict is absent. Operating with a patchwork of isolated frames, which do not yield contradictory results, is sufficient for developing a consistent, stable mathematical domain, without exhibiting the essence of modern, formal, global approaches implicitly. The cognitive conflict cannot arise in this situation as there is no metamathematical requirement in opposition with the conceptual frame, nor can the cognitive conflict be considered tacit within the frame as there are no ‘hidden tensions’ awaiting resolution as Desargues’ projective geometry is consistent—this is not an elision, but rather an absence. The oppositional elements giving rise to the cognitive conflict are introduced through the addition of metamathematical requirements at odds with the conceptual frame, namely the requirement that inference must occur within global unitary mode and that there be one unique point at infinity, with the resultant change in conceptual frame away from directed line segments, as seen in Coxeter.

### Jean Victor Poncelet (1788–1867)

#### The introduction of the point at infinity

Jean Victor Poncelet published his *Traité des propriétés projectives des figures* in 1822, nearly two hundred years after Desargues’ work, and published a second edition in (1865). He produced the substance of the work while a prisoner after the Napoleonic wars in which he had served as an engineer. During this time, Poncelet rederived and expanded much of Desargues’ work. He mentions that Desargues had a custom to

‘considérer les systèmes de droites parallèles comme concourant à l’infini, et qu’il appliquait le même raisonnement qu’aux lignes convergentes’ (1865, p. xxvii)

*‘consider systems of parallel lines as concurrent*^17^*at infinity, and that he applied the same reasoning as to convergent lines’* (Our translation)

Not only does this evidence his acknowledgement of the prior work of Desargues, but this also serves as his introduction to the point at infinity. As with Desargues, Poncelet offers little by way of explanation as to the meaning of the point at infinity, although there is an instruction to treat parallel lines as if they were convergent lines, converging at infinity. The cognitive conflict and the question it raises, such as how the two intuitive points at infinity can be one, are not afforded even passing mention. Much like Desargues there is no evidence in Poncelet’s introduction that the cognitive conflict arose, or was at least considered important enough to put into writing.

#### The use of the point at infinity in inference

After some time spent describing what it means for a property to be projective, and demonstrating some such properties, Poncelet begins to consider figures in which a previously finite point is now at infinity.

**Fig. 6:**
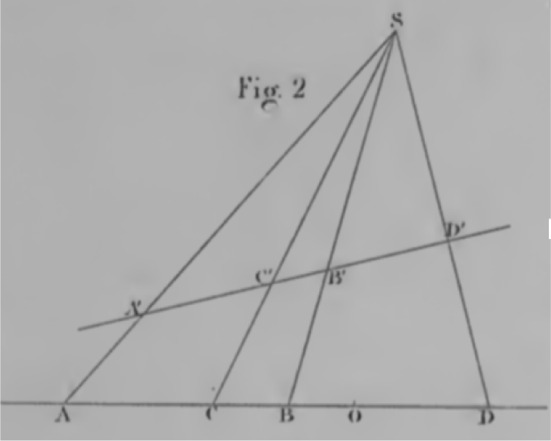
Poncelet *fig. 2*

‘Supposons *(fig. 2)* [Fig. 6] que le point D soit à l’infini, ou que SD soit parallèle à AB; les segments DA et DB devenant à la fois infinis’ (1865, p. 14)

*‘Let us suppose (fig. 2) * [* Fig. *6] *that the point D were at infinity, or that SD were parallel to AB; the segments DA and DB becoming at the same time infinite’* (Our translation)

The figure (Fig. 6) and the description of the point *A* as at infinity or the line *SD* as parallel to *AB*, both in the subjunctive tense with the verb être, *to be*, are both static depictions of the scenario—departing from Desargues’ dynamic, directed, arboreal language. In fact, note that the grammatical form for reference of a point being at infinity is the same as that for a line being parallel. This indicates that the background space in which Poncelet’s figures are situated has a pre-existing location for a point at infinity in the same way that it has a location in which a parallel line could be. This is a contrast to Desargues, where a point could only become infinitely removed through the extension of a line segment. Perhaps then, Poncelet is invoking a different inferential structure, one closer to and perhaps implicitly using our modern formal constructions.

**Fig. 7:**
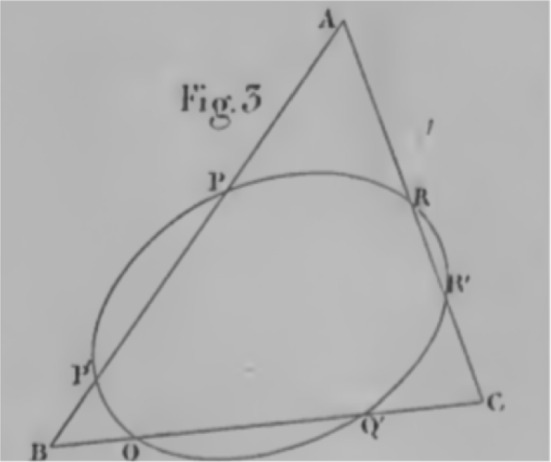
Poncelet *fig. 3*

Note that the line segments in the previous quote and Fig. 6 are not simply mentioned in alphabetical order. We see ‘*AB*’, but also ‘*SD*’, ‘*DA*’, ‘*DB*’. This reversal of alphabetical order is not an idiosyncratic quirk, but a reflection that the order of the labels reflects an ordering of the endpoints of the lines, as we saw in Courant and Robbins. The line segments defined by a pair of points has a direction, starting from the textually first point, say *S*, and ending at the textually second point, say *D*. When describing the segments *DA* and *DB*, Poncelet does not refer to them in the subjunctive tense with the verb être as he does with the point *A* and the line *SD*, instead he refers to them with the present participle ‘devenant à la fois infinis’: *becoming at the same time infinite*. The use of the present participle, and the verb ‘devenir’—to become—invokes a frame in which there is an event that occurs through a period of time, an inherently dynamic frame. This role of fictive motion, combined with the nominal ordering of points when referencing a line segment, strongly suggests a role of direction, as per Desargues, even with the absence of an explicit grounding in Directed Line Segment Frame. Although the grounding in extending line segments appears to have been the seminal conceptual frame, invoking frames that rely on direction without the extension feature could still allow for the local ad hoc treatment of the point at infinity.

**Fig. 8:**
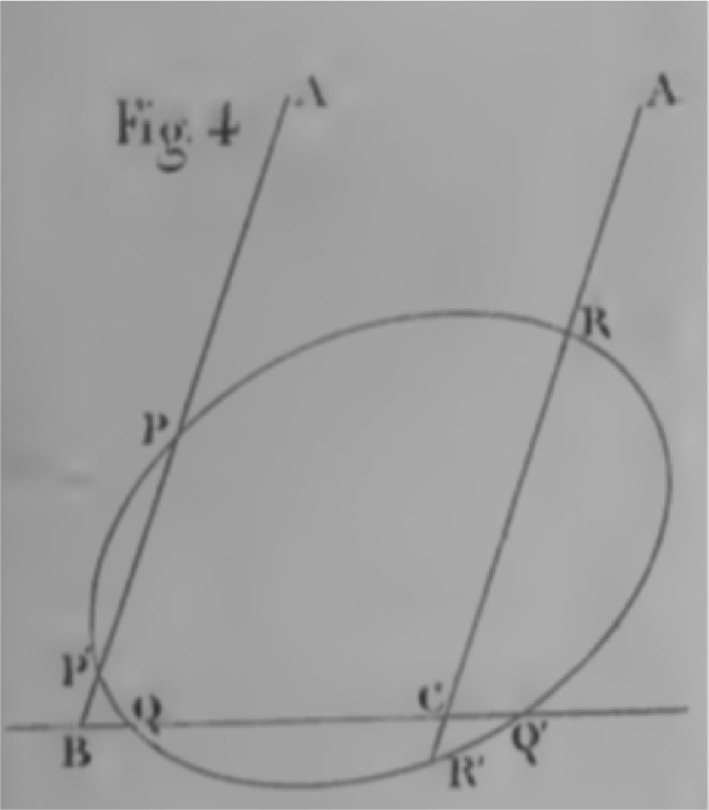
Poncelet *fig. 4*

In Figs. 7 and 8, we see further evidence of Poncelet’s invocation of direction and motion. Having discussed Fig. 7 Poncelet considers Fig. 8 within that context,

‘Supposons que les côtés AB et AC, de concourants qu’ils étaient, deviennent parallèles *fig. 4* [Fig. 8], le sommet A passera à l’infini’ (1865 , p. 18)

*‘Let us suppose that the sides AB and AC, that they were concurrent*^18^, *were*^19^*to become parallel *[*Fig.*  8]*, the vertex A will go to infinity’* (Our translation)

In this case, the subjunctive tense with the verb ‘devenir’, *to become*, deployed to express the hypothetical case that the two sides *AB* and *AC* became parallel. Rather than simply stating, as he did in the previous case—‘que le côté AB soit parallè à AC’, supposing that they were both parallel—Poncelet explicitly invokes a frame in which the current configuration is transformed, through time, to the position in which the lines are parallel. This frame then entails that the vertex *A* move in one direction or another. If the lines were simply stated to be parallel, then the question of where to represent *A*, at which end of the line or both ends of the line, would present itself. However, by invoking a frame in which the lines become parallel, this avoids that cognitive conflict. For the lines to become parallel, one must rotate in a consistent direction, say clockwise, relative to the other. This requirement ensures that the point A recedes towards infinity in only one direction prior to the lines becoming parallel. We see further evidence that Poncelet is invoking this frame in the actual depiction of his *fig. 4* (Fig. 8), where the line segments *AB* and *AC* are only extended in one direction. This restriction is not due to Poncelet refusing to typographically represent a point in two distinct locations, as he does so for this point A—marking it doubly, once at the ‘top’ of each of the lines. Thus, his representation of only one half of the line stems from invoking frames in which direction is an inherent aspect.

From Poncelet we can see that the cognitive conflict with which we are concerned does not require a resolution, even if the point(s) at infinity are independent of a construction by the extension of a line segment. Desargues was concerned with practical constructions to which his theoretical geometry served, and his work was thus grounded in Directed Line Segment Frames. Poncelet was less practical in his aims and was instead interested in configurations of geometric elements and their properties. However, in order to reason about these configurations Poncelet used his *principle of continuity*. Poncelet relied on transformations from configurations about which theorems were known, to configurations (often involving parallel lines that were previously concurrent) about which the theorems were uncertain. Despite these two very different problem situations, the frames invoked in inference entail or rely on a consistent direction for locally cohesive inquiries and problems, and thus the issue of two distinct points being identified is not a mathematical problem—or at least is not a problem that these eminent mathematicians deem worth addressing, or that presents itself in inferential practice.

In sum, for both Poncelet and Desargues, the cognitive conflict is absent as the constitutive components are missing, but the inferential structure remains cohesive and contains all the projective geometric truth statements. Invoking directed frames in their manner omits from the practice the global unitary mode—one of the essential opposing elements out of which the cognitive conflict arises—and allows for consistent, stable reasoning in a local ad hoc mode, and the mathematical problems and inquiries are made local by the invocation of these isolated, directed frames.

## Discussion

In modern mathematics, the real projective plane is formally constructed by a *stipulative approach* or a *gluing approach*, yielding a formal system in which the point at infinity is defined such that it is globally applicable in inference independently of the context through which it arises, as evidenced by the reference texts. All possible representations of the point at infinity, or differing points at infinity, are constructed and shown to be substitutable for one another within inferential practice, or there is a semantic shift away from treating points as locations to viewing points as primitive formal mathematical objects. The formal system has a global unitary construction of the point at infinity which serves a specific goal: to satisfy modern metamathematical views as to how inference should occur—the global unitary mode—and to avoid exceptions and generalise Euclidean geometry.

This global unitary construction could have been present or implicit in earlier work. There could have been an appreciation by pre-modern geometers of the cognitive conflict central to projective geometry, and periods of struggle to grapple with it. There might have been extensive discussions of how to reckon with two seemingly incompatible points at infinity that must be one, or an awareness that different frames imply different points at infinity. In this hypothetical historical period we may have been able to claim that there was a ‘common thread’ that lay ‘hidden’ between the modern and pre-modern approaches, as formulated by standard arguments for mathematical realism. However, we see no evidence for this in these seminal texts. Curiously, there is no evidence that this cognitive conflict ever presented itself to these seminal authors. Their introductions of the point at infinity are short, and whenever the point at infinity is present in inference the cognitive conflict is absent due to a reliance on isolated, directed frames. Desargues’ approach grounds the discussion in the frame of extending line segments. Similarly Poncelet, although moving away from line segment extensions, relies on directed frames via his manner of invoking fictive motion of lines and associated points of intersection. Both of these seminal authors invoke directed frames within which inferences concerning the point at infinity are ad hoc and considered locally, isolated from other frames. In no directed frame could both possible points at infinity be inferentially relevant. Their projective geometry relies on a mutually consistent patchwork of isolated local frames. Necessary components of the cognitive conflict are absent in this local ad hoc mode of inference, and thus the cognitive conflict cannot arise—the cognitive conflict is neither resolved nor elided by these seminal authors.

The patchwork of isolated frames is conceptually incompatible with constructions in the modern, global unitary mode. This is best evidenced by considering the comparison of Courant and Robbins who implicitly invoke a dynamic, local ad hoc mode, and Coxeter who implicitly invokes a dynamic, global unitary mode. Courant and Robbins invoke fictive motion in local directed frames. These local frames construct the point at infinity in an ad hoc fashion by parameterising the BMI with, and within, these directed frames. This approach renders the cognitive conflict of the point at infinity meaningless in practice. Inference proceeds consistently by operating within each of the local frames individually, and through agreements among the patchwork frames. This is in stark contrast to Coxeter. He too invokes fictive motion, however, he persists with the global character of his *stipulative approach*. With this approach, statements concerning points moving *through* infinity are rendered meaningful. For Coxeter a point can move from one side of infinity to the other. It is not possible to evaluate this statement in Courant and Robbins’ conceptual framing. For in the latter, a key feature of the concept of Through is lacking. There is nothing beyond the point at infinity. For both sets of authors there are points immediately to the left and immediately to the right of the point at infinity. However for Courant and Robbins these elements occur in separate conceptual frames, while for Coxeter they occur within the same frame, thus Throughness is only meaningful in the latter’s construction. These differences are not captured in the truth-statements of the object-language, their theorems are the same.

One might contend that the differences between Courant and Robbins and Coxeter are not sufficient to support the claim that their inferential structures are conceptually incompatible. However, unlike Benacerraf’s (1965) identification problem, the differences between these inferential structures are not simply in the formal meta-language, but in the composition of the inferential structures. The local ad hoc mode relies on a patchwork of isolated conceptual frames in which inference is conducted and then compared across frames for well-definedness. One cannot move between frames the way one can switch and swap representations in modern mathematics, especially not with regard to the point at infinity: its role in inference is dependent on the frame in which it is constructed ad hoc.^20^ Not only can different points at infinity not substitute for one another, there is only ever one frame-relevant point at infinity—all others are meaningless within that frame. This is conceptually incompatible with the frames and methods in the global unitary mode. In this modern mode one considers the multitude of different points at infinity within one monolithic frame, and then develops formal tools for reckoning with this cognitive conflict resulting in the ability to substitute such points for one another without affecting inference (or by resorting to purely formal, syntactic modes of inference). These two modes cannot be reconciled—at least not by methods currently popular in the philosophy of mathematics. Further, there is no evidence that the seminal authors ever considered such a multitude of points—in which case one might be able to substantiate the claim of ‘common thread’. Their specific practices never introduced such an issue, as necessary elements constituting the cognitive conflict were absent in their work and grounding.

It is a matter of contingent fact that we do not find contradictions either in Desargues’ or Poncelet’s inferential systems, nor in the local ad hoc mode implicit in Courant and Robbins. There are no ‘hidden tensions’^21^ that after much inspection required resolution. The global unitary mode, with a monolithic frame exemplified by the reference texts and Coxeter cannot be considered the explication of endogenous issues within the domain for there were no such issues to be resolved. This case of development requires a different analysis than those driven by ‘hidden tensions’, and appears to support the argument that some mathematical domains have been subject to revision as a result of exogenous metamathematical shifts from which conceptually incompatible inferential structures emerge that cannot be considered explications of prior systems. Philosophies of mathematics that rely on historical accounts of progress in support of mathematical realism must be adjusted, abandoned, or restricted to accommodate cases of this sort.

## Conclusion

Using the tools of cognitive semantics and linguistics, broadly understood, we have found that there are conceptually incompatible means by which the projective geometry can be structured, constructions adhering to either local ad hoc or global unitary modes, and that these differences appear in the historical development of the subject. The features that distinguish these constructions are not contained in the truth statements of the object-language. Although constructions in the global mode emerge historically later, and appear to model statements that constructions in local mode do not, they are in fact mathematically equivalent and it is not clear that we should consider those of the global mode as refinements of those in the local mode. If one wishes to make this claim, one needs to articulate precise desiderata that an inferential structure must satisfy in order to be an implicit version of another. Historians and philosophers may be correct that there are certain paradigms of mathematical development in which the later formulations truly capture the ‘common thread’ that lay ‘hidden’ and thus explain ‘why those [earlier solutions] worked’. In this case however, given the stark contrast between the two local systems of Desargues and Poncelet and the global mode immanent in the normative modern formulations, it appears that such claims are not consistent with this analysis, and that to consider that

‘Kepler (and Desargues) regarded the two “ends” of the [projective] line as meeting at “infinity”, so that the line has the structure of a circle’ (Kline, 1972, p. 290)

is simply untenable given the evidence we have presented here.

Linguistics broadly understood, and cognitive linguistics in particular, is necessary to construct valid philosophies of mathematics. The features distinguishing the two systems of projective geometry—local ad hoc and global unitary—are not evident in the truth statements of the object-language. These two systems persist in modern textbooks, and were deployed in different periods in mathematical development. The change from one system to the other cannot have been the result of ‘hidden tensions’ nor can we appreciate the change by focusing on ‘truth reversals’. We need a diversity of tools to inform the philosophy and history of mathematics. The desiderata for claims of implicit or hidden concepts must be constructed in the context of the information that can be gained from both formal and cognitive linguistic analyses. Cognitive linguistic analyses must continue to be performed in all considerations of mathematical progression. The application of formal tools of analysis to mathematical progress and development contributes much to the philosophy of mathematics, but without expanding the toolset to include cognitive linguistics—frame semantics, tense, conceptual mappings, fictive motion, diagrams—and further to situated practice—temporally constructed sketches, gesture production, gaze etc.—much of the important data concerning mathematical practice and progress will be rendered invisible. Our histories and philosophies will be under-constrained, and leave us with grand, untenable narratives of the unique determination of mathematical progress.

## Footnotes

1 The *no-miracles argument* contends that the only position that does not make the success of science, or mathematics, a miracle is the realist position. It is an inference to the best explanation, predicated on accounts of historical change and progress.

2 We will not use this term to refer to structures as abstract entities, but rather as means of performing inference. These means could be formal, cognitive, material etc. or the combinations thereof. That is, the inferential structures are theoretical constructs for describing the practical means by which mathematicians perform inference without any particular form being privileged.

3 Following a convention in cognitive linguistics, we denote specific concepts with small capital letters (e.g. Mother) and use lower case letters to refer to linguistic expressions that may use these concepts e.g. ‘your mother was born in Australia’ and ‘mother nature is wise’.

4 The conceptual blend involves further linguistic details such as the clashing of the perfective and imperfective aspect in the first and second input space. For details see (Núñez, 2005).

5 As noted in the introduction, rather than an abstract or formal entity, an inferential structure is a theoretical description of a means of performing inference. The blended space is here a specific instantiation of an inferential structure, but by no means the only one.

6 This is not the only possible such cognitive construction. There could be others. We can examine which semantic structures are invoked to ground the meaning of this specific infinity by examining the verbs, tenses, typographics, diagrams (and outside of a text: e.g., the gestures produced by the speakers etc.) thus distinguishing different groundings from one another.

7 Debates as to the source of meaning, and its stability, are not settled within the philosophy of mathematics (see for instance Pérez-Escobar & Sarikaya, 2022). We are not taking a specific stance here, but hold the position that for a cognitive agent operating with a frame obliges certain inference—entailments. For instance, suppose one operates with the widely spread frame in which the future is conceived as being in front of the ego (i.e. the speaker), the past is behind the ego, and extent in time is mapped to extent in saggital space with a transitive relation in time mapped to the same in space. Then an entailment of that system is that, if discussing the upcoming week on a given Monday, Friday is a greater distance in front of the ego than Wednesday—as is exhibited in co-speech gesture (Núñez & Cooperrider, 2013).

8 Ad hoc is a Latin phrase that literally means ‘to this’. Many European languages have borrowed the expression in accord with the definitions given in the text. However, in English the expression can often convey negative connotations of ill-preparedness. To be clear, in this article we invoke *only* the term’s standard definition as seen in dictionaries to capture the frame-dependent construction of the point at infinity in some of the texts in our case-study. It is only in the context of the frame, and a particular problem at hand, that the construction is meaningful, and it is only on the basis of the background frame and our general cognitive mechanisms that such a point is constructed.

9 We do not prescribe all the manners by which the move to the global, unitary mode can occur. This could be achieved by a new conceptual foundation, or via formal methods that break the inferential isolation of the separate frames, or maybe constructing a super-ordinate frame. All of these result in the same effect, thus raising the cognitive conflict.

10 This explanation is equivalent to the CW-complex construction of $\mathbb {R}P^2$. For purposes of space we suppress the complete explanation here, however see nLab (2022b). It also shares properties with the construction of $\mathbb {R}P^2$ as the sphere ‘but with the significant difference that opposite points are regarded as the same’ (Gowers et al., 2008, p. 43).

11 This does not necessarily mean that practicing mathematicians come to enact some new, monolithic conceptual frame (though repeated use of the gluing approach might eventually encourage such). It simply provides a method for stable inference in light of the oppositional elements that constitute the cognitive conflict.

12 Apart from the ‘line at infinity’ which contains all the ‘points at infinity’ but no finite points.

13 This does not deny that the latter was inspired by the former.

14 Strictly speaking neither a mathematical line, nor a mathematical point, can move. A point X is either a specific co-ordinate location or a synthetic object. A point cannot move and retain its identity the way that physical objects can. If a point is at a new location, or is a new synthetic element, then it is no longer the original point—its identity has changed. This precludes movement as movement is predicated on identity-constancy. The same holds for lines, which are sets of points: if a line rotates it consists of new points, it is therefore a different set, a different line.

15 See for instance the language deployed in describing perspectivities and their relationship to the point at infinity (1916, pp. 7–14).

16 A skeptic might here note that in modern mathematics limits and continuity are grounded by formal $\epsilon -\delta$ definitions which invoke a static frame and the preservation of closeness. However, rendering this case-study into $\epsilon -\delta$ definitions does not resolve the choice issue nor does it capture the essence of the inferential structure in the directed, dynamic frames. Further, modern mathematicians continue to invoke dynamic frames in practice, even when proving formally, see Marghetis and Núñez (2013). In this case-study, and the choice issue in the counterfactual, we see what Núñez described as the fact that ‘what is characterised formally in mathematics leaves out a huge amount of inferential organisation of the human ideas that constitute mathematics’ (2006, p. 168).

17 In modern mathematics ‘concourant’ means convergent. However, given that the term ‘convergentes’ is used later in this same quote, we feel that ‘concurrent’ is a more accurate translation, and highlights the difference between Poncelet’s conception of the concurrence of parallel lines and the convergence of skew lines.

18 In English the imperfect past tense of ‘qu’ils étaient’ is suppressed, and cannot be distinguished from the simple past. The imperfect past refers to an event or state that was ongoing, which is important in the context of identifying or denying the presence of fictive motion. For this reason, we have offered a non-standard English translation to highlight this imperfective aspect.

19 The word ‘deviennent’ is the third person plural conjugation of ‘devenir’ in both the indicative and subjunctive present tense. Given the context of the preceding imperfect past state of the lines, the dynamic verb ‘passer’ in the future tense, we feel the subjunctive translation is appropriate. However, in this case the resulting analysis does not change whether the indicative present translation is instead accepted.

20 This inability to switch between frames is not a cognitive limitation, but is the result of some terms of one frame being meaningless in another, such that questions across frames are ineffable.

21 In Poncelet, one might argue that as he had cause to rotate concurrent lines into a parallel position that perhaps rotation through the parallel (and thus the global character evidenced in Coxeter) was in fact a ‘hidden tension’ in his work. We do not have space to give this full consideration here. However, given his interest in static configurations and use of rotations as a method for extending results rather than as the object of inquiry, it is not immediately obvious how one should consider this with respect to ‘hidden tensions’. If one can satisfactorily demonstrate that this is a ‘hidden tension’, this line of reasoning will not extend to Desargues’ work with its drastically different grounding, and thus our point concerning continuity still stands. In either case, our general aim to evidence the need and use of cognitive linguistics to inform the philosophy of mathematics remains.
